# Histological Characterization of the Irritative Zones in Focal Cortical Dysplasia Using a Preclinical Rat Model

**DOI:** 10.3389/fncel.2018.00052

**Published:** 2018-05-18

**Authors:** Abhay Deshmukh, Jared Leichner, Jihye Bae, Yinchen Song, Pedro A. Valdés-Hernández, Wei-Chiang Lin, Jorge J. Riera

**Affiliations:** Neuronal Mass Dynamics Laboratory, Department of Biomedical Engineering, Florida International University, Miami, FL, United States

**Keywords:** epileptogenesis, chronic seizures, irritative zones, histology, brain source imaging

## Abstract

Current clinical practice in focal epilepsy involves brain source imaging (BSI) to localize brain areas where from interictal epileptiform discharges (IEDs) emerge. These areas, named *irritative zones*, have been useful to define candidate seizures-onset zones during pre-surgical workup. Since human histological data are mostly available from final resected zones, systematic studies characterizing pathophysiological mechanisms and abnormal molecular/cellular substrates in irritative zones—independent of them being epileptogenic—are challenging. Combining BSI and histological analysis from all types of irritative zones is only possible through the use of preclinical animal models. Here, we recorded 32-channel spontaneous electroencephalographic data from rats that have focal cortical dysplasia (FCD) and chronic seizures. BSI for different IED subtypes was performed using the methodology presented in Bae et al. (2015). Post-mortem brain sections containing irritative zones were stained to quantify anatomical, functional, and inflammatory biomarkers specific for epileptogenesis, and the results were compared with those obtained using the contralateral healthy brain tissue. We found abnormal anatomical structures in all irritative zones (i.e., larger neuronal processes, glioreactivity, and vascular cuffing) and larger expressions for neurotransmission (i.e., NR2B) and inflammation (i.e., ILβ1, TNFα and HMGB1). We conclude that irritative zones in this rat preclinical model of FCD comprise abnormal tissues disregarding whether they are actually involved in icto-genesis or not. We hypothesize that seizure perpetuation happens gradually; hence, our results could support the use of IED-based BSI for the early diagnosis and preventive treatment of potential epileptic foci. Further verifications in humans are yet needed.

## Introduction

The primary aim of the pre-surgical evaluation of patients with refractory focal epilepsy is to define those brain areas responsible for seizure generation. Brain source imaging (BSI) of the interictal epileptiform discharges (IEDs) is the most widely used method to localize these epileptogenic zones (Kaiboriboon et al., 2012). The BSI methodology in epilepsy consists of first classifying IEDs into spike and sharp-wave types (Tyvaert et al., 2008) from electroencephalographic (EEG) data, followed by solving the EEG inverse problem to localize their brain generators, named *irritative zones*. In practice, many irritative zones are usually found due to the existence of either multiple types of IEDs (Pedreira et al., 2014) or single IEDs with widely distributed current sources (Heers et al., 2015). Nevertheless, only a few irritative zones showing concordance with data from other neuroimaging modalities (e.g., inter-ictal PET, ictal SPECT, and functional/anatomical MRI) and in agreement with the seizure semiology are considered of clinical relevance, and therefore discussed during the pre-surgical workup.

Irritative zones not discussed in the initial pre-surgical workups are only reconsidered for cases with negative post-surgical outcomes, i.e., “Engel Classification.” Large variations in seizure recurrence timing after surgery suggests dynamic changes in tissue properties. We hypothesize that abnormalities in the molecular/cellular substrates in most irritative zones could eventually prompt some of them to seizure genesis during neurodevelopment and after surgical procedures; hence, they need to be properly characterized. Undergoing inflammatory processes, triggered by either seizures or lesions, might cause local changes in gene expression, and consequently in tissue composition, facilitating increases in neuronal hyperexcitability, glio-reactivity and angiogenesis in most irritative zones. Although a longitudinal study will be needed to test this hypothesis, an evaluation of the existence of important biomarkers for epileptogenecity in irritative zones at a given time-point will provide insights into potential pathways for seizure recurrence and preventative therapies. Several molecular/cellular biomarkers have been associated with epileptogenesis in focal cortical dysplasia (FCD), one of the most abundant types of refractory epilepsy in children and associated with lesion-like malformation of cortical development (Sisodiya et al., 2009). For example, an up-regulation of NMDA (NR2B) receptors in type II FCD has been reported in previous studies for humans (Crino et al., 2001; Finardi et al., 2013) and rats (Colciaghi et al., 2011). Alterations in the GABAA-receptor-mediated inhibition (Crino et al., 2001; Calcagnotto et al., 2005) and in mGluR5 receptor expression (Aronica et al., 2003) for dysplastic regions have been also confirmed in the past. Large dysmorphic neurons characterized by cytoplasmic accumulations of neurofilaments have been extensively found in these lesions (Thom et al., 2005; Finardi et al., 2013). Reduced neuronal cell density (Thom et al., 2005; Finardi et al., 2013) and extensive reactive gliosis (Rasmussen et al., 1958; Devinsky et al., 2013; Finardi et al., 2013; Kielbinski et al., 2016) have been previously described in dysplastic areas. Changes in these particular cellular distributions have been correlated with the duration of epilepsy (Finardi et al., 2013). Finally, there are several recent preclinical/clinical studies linking epileptogenesis, in particular for dysplastic brain regions, with neuroinflammatory IL-1β, TNF-α, and HMGB1 signaling (Vezzani and Granata, 2005; Maroso et al., 2010; Zurolo et al., 2011; Vezzani et al., 2013).

Unfortunately, it is challenging to perform a systematic evaluation of the molecular/cellular composition in pre-epileptogenic irritative zones using humans due to the fact that resected tissues are only available from brain areas associated with seizure genesis. Therefore, preclinical animal models are essential to study such cellular/molecular abnormalities that could potentially prompt irritative zones to icto-genesis. A MAM-PILO double-hit model for FCD and chronic seizures on Wistar rats (Colciaghi et al., 2011) have been successfully used in our laboratory to study different physiological mechanisms associated with FCD (Bae et al., 2015; Song et al., 2015, 2016). Technical difficulties associated with the performance of BSI on rodents, the inferior mammalian used typically to study epilepsy (Löscher, 1999), have been recently solved with the introduction of an EEG mini-cap (Bae et al., 2015; Riera et al., 2015) and the development of a theoretical construct to solve small-scale forward/inverse problems (Valdés-Hernández et al., 2016).

In this study, we aim at finding long-term IED-induced molecular/cellular changes in the irritative zones of this particular rat model of FCD. To accurately classify multiple IEDs from actual EEG data, we combined a three-way principal component analysis (parallel factor analysis, PARAFAC) (Acar et al., 2007) and a cluster analysis (Pedreira et al., 2014). Source localization of the individual IEDs subtypes was performed using the sLORETA method (Pascual-Marqui, 2002). Local field potentials (LFP) were used to assess whether some irritative zones were actually epileptogenic or not (Song et al., 2016). A Wistar rat T2-weighted probabilistic atlas (Valdés-Hernández et al., 2011) was used to precisely determine from the BSI analysis the brain blocks for sectioning. For each irritative zone, we used histological biomarkers to evaluate anatomical (i.e., neurofilaments and neuronal cell density), functional (i.e., NR2B, GABAA and mGluR-5 receptors), and inflammatory (IL-1β, TNF-α, and HMGB1 signaling) abnormalities known to be involved in epileptogenesis in cases of FCD.

## Materials and Methods

### Animal Preparation

The pharmaco-resistant FCD preclinical model was established using the protocol described in Song et al. (2015, 2016). In brief, this protocol includes two major steps. First, cortical malformations were induced prenatally on Embryonic Day 15 with two doses of methylazoxymethanol (MAM) injections (15 mg/kg maternal body weight) 12 h apart. This procedure was performed at Charles River Laboratories. Second, MAM-treated rats were intra-peritoneally (i.p.) injected with pilocarpine (PILO, 300 mg/kg) on post-natal day 28 to induce status epilepticus (SE). All MAM-PILO rats were monitored by four investigators independently to identify the SE onset. According to the Racine scale (Racine, 1972), a rat was considered in SE when having continuous rearing/falling, and sporadic motor seizures (stage 5). Rats experiencing SE received intra-peritoneal phenobarbital (20 mg/kg) 90 min after SE onset to reduce mortality, then were hydrated subcutaneously with Lactate Ringer’s solution and fed by the investigators to improve the survival rate. Out of 62 male Wistar rats used for this study, 26% rats survived this procedure. Rats not experiencing SE were not considered and euthanized. After 2 weeks from onset of SE, rats were observed for occurrence of seizures for 1 week to confirm the chronic epileptic models. Eventually, 13 MAM-PILO rats developed chronic seizures. From this point on, these MAM-PILO rats will be referred to as epileptic rats. Data from 6 epileptic rats were not used because one of the following reasons: (a) either due to inconclusive results from the BSI analysis or not having a clear contralateral healthy control (3 rats), failures during perfusion (1 rat), or incomplete/missing data (2 rats). Therefore, all statistical analyses presented in this paper were performed using seven rats, with one having two clear non-overlapping irritative zones for which the respective contralateral tissues were healthy. We included each of these two irritative zones for this particular rat as additional regions of interest, resulting in eight irritative zones over 7 rats (*N* = 8). The proposed method is assumed valid since the variability level across cortical regions is larger than that across rats (same cortical region). In our experiment, the regions of interest are randomly located in different cortical areas for each rat resulting from the nature of the epilepsy induction mechanism. Furthermore, each irritative zone, including the two for this particular rat, contains independent local cortical architecture. Finally, the paired-sample design used in this study will account for any further inhomogeneity in the variance level. All epileptic rats were housed in standardized cages at a 12 h-12 h light-dark cycle with food and water ad libitum.

### Electrophysiological Recording

#### For EEG Recordings

**FIGURE 1:**
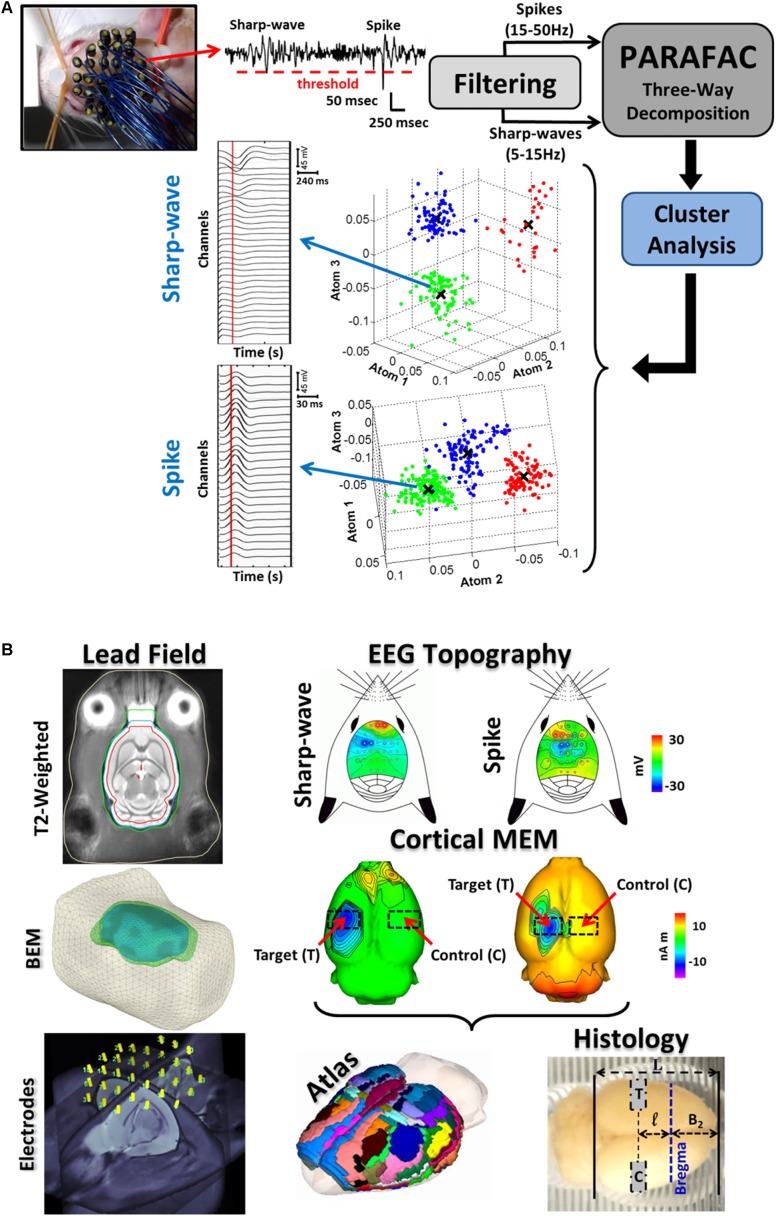
Illustration of the entire methodology for the IED-based brain source imaging (BSI) and immuno-histology. **(A)** 32-channel EEG recording were obtained with the EEG-mini cap. A thresholding method (red-dashed horizontal line) was used to detect the IEDs from the 32-channel scalp EEG (top-left, a single channel is illustrated). IED spike (20–70 ms in duration) and sharp-wave (70–200 ms in duration) waveforms were separated by means of multiple band-pass/notch filters (*spikes*: 15–50 Hz and *sharp-waves*: 5–15 Hz). Both signals were classified into subtypes by combining the parallel factor analysis (PARAFAC) and the k-mean cluster method. PARAFAC allows decomposing the topographic maps and temporal courses of IED subtypes as combination of spatial and temporal atoms, reducing uncertainty in IED classification. The atom-based 3D plots for both spikes and sharp-waves represent separable subtypes (clouds of different colors). Note that each dimension of our three-way data represents space (channels), time, and sample (IEDs). Each subtype of IEDs was considered as a unique epileptiform event. Mean time courses for each IED subtype were obtained by averaging over samples of the same cluster (i.e., mean value **X**). **(B)** For each rat, electrodes on the EEG mini-cap were co-registered to our T2-weighted probabilistic atlas and template. Segmentations of different surfaces (e.g., cortex, inner/outer skull and scalp) of this T2-weigthed template image were used in the boundary element method (BEM) to estimate the lead field matrix needed to solve the EEG inverse problem for each rat. IED source localization is believed to be more accurate at the 2/3 of the IED peak (vertical red lines in the IED average time series). The corresponding IED topographic map for each subtype reveals localized cortical activation in most of the cases. sLORETA inverse solution shows the specific cortical source locations and its contralateral non-activated counterpart (red arrows). Cortical current generators for each IED subtype were co-registered with the T2-weighted probabilistic atlas using procedures described in Bae et al. (2015). The cortical regions with maxima current amplitudes in the 2/3 initial phase of each IED were selected as target areas (T), and the corresponding contra-lateral healthy regions as controls (C). The co-registered sLORELA inverse solution (left) is in the Paxinos-Watson’s coordinate system, so actual cortical regions can be identified according to the convention in **Table 2** (Atlas, middle panel). Brains were scaled according to the formula in Section “Materials and Methods” and blocks for histology were cut using a scaled brain matrix (right).

Rats were fixed on a stereotaxic apparatus under deep anesthesia (isoflurane 1.5–2.5%, 1 L/min O_2_, 14.7 PSI). The EEG mini-cap was set on the rat’s head following the procedure described in previous studies (Sumiyoshi et al., 2011; Bae et al., 2015) (**Figure 1A**, *picture*). Dexdomitor (dexmedetomidine hydrochloride, 0.25 mg/kg) was administrated via i.p. injection and then isoflurane was gradually reduced to 0%. Dexdomitor does not reduce IED rate (Song et al., 2015, 2016). EEG ongoing activity was recorded (PZ3, Tucker-Davis Technology, TDT) for periods of 30 min. EEG (0.5–250 Hz) signals were filtered and digitized (0.5 μV resolution) using the OpenX software (TDT). The heart (200–300 BPM) and respiration (<50 BPM) rates were continuously monitored using the PowerLab 8/35 data acquisition device and the LabChart software (AD Instruments). These data were used to monitor the level of anesthesia and rat’s physiology throughout the entire experimental procedures. Temperature was maintained (37°C) using a water-circulating heating pad (TPZ-0510EA, Texas Scientific Instruments) with a pump (TP700, Texas Scientific Instruments).

#### For LFP Recordings

Six out of the seven rats (**Table 1**) underwent brain surgery for intracranial recording. A craniotomy (2 mm) was performed on top of one of the irritative zones as described in Song et al. (2016). For each epileptic rat, the surgically accessible location for craniotomy was determined using BSI (Bae et al., 2015). A silicon-based probe (A16, 16-channel linear probe, 100-μm intervals, NeuroNexus Technologies) was inserted perpendicular to the cortex. In this particular case, dexdomitor (0.25 mg/kg, i.p.) was combined with low doses of isoflurane (0.5–0.8%, 1 L/min O_2_, 14.7 PSI). Long periods (2 h) of LFP recording were used to determine whether or not a particular irritative zone generates seizures. These rats have several seizures per day, especially during the period recorded (5pm - 7pm). LFP recording was performed with amplifiers at 25 kHz (PZ2, Tucker-Davis Technologies, “TDT”). Rat brains were perfused at the end of the LFP recordings. Therefore, we expect no inflammatory responses due to the insertion of the electrophysiological probe, whose size was very small (15 μm × 123 μm). Brain slices containing any signature of a probe insertion were not included in the histological analysis. LFP (0.7–170 Hz) signals were filtered and digitized (0.5 μV resolution) using the OpenX software (TDT). LFPs recorded from these six rats were also used in our previous study (Song et al., 2016). Some results from our BSI analysis were also presented in Bae et al. (2015).

**Table 1:** This table shows the numbers of classified averaged spikes (S) and sharp-waves (SW) for each rat, which were used separately in the BSI methodology.

Rat	IEDs		Localized structures	Most frequent structure *Target area (Histology)*	Craniotomy	Seizures-onset During LFP recording
					
	*S*	SW				
1882	3	3	67, 6, 56, 50, 52, 59, 80, 84, 63, 5, 60, 34, 12, 82, 72	67 **M1** - *left*	**V1M** - *right*	No
1884	4	3	20, 68, 82, 50, 52, 19, 60, 6, 67, 84	20 **M2** - *right*	N/A	N/A
2225	4	5	79, 82, 52, 87, 67, 80, 88, 19, 20, 68, 6, 34, 43, 12, 15, 53, 54	79 **S1BF** - *left*	**S1HL** - *left*	No
2223	2	4	43, 75, 95, 76, 67, 12, 27, 14, 31, 2, 32, 69, 94, 92	43 **V1B** - *right*	**V1B** - *right*	No
1888	2	2	47, 80, 82, 34, 79, 12, 14, 36, 84, 32, 33	47 **V2MM** - *right* 80 **S1DZ** - *left*	**M1** - *left*	Yes
1894	3	3	46, 39, 19, 7, 12, 96, 5, 45, 6, 40, 35, 20, 69, 31, 14, 55, 89, 43, 44	46 **V2ML** - *right*	**M1** - *left*	Yes
1900	3	3	32, 52, 34, 12, 68, 54, 69, 19, 79, 53, 96, 15, 6, 14, 5	32 **S1DZ** - *right*	**AuD** - *right*	Yes


*Each spike and sharp-wave caused activation of different cortical structures represented by assigned numbers which are defined in the **Table 2**. Structures are listed as most frequent to least frequent, accordingly. The most frequently localized structure, with an overall inactive contra-lateral region, was considered as the target irritative region for each rat and used for further histological analysis. Two target areas were found for rat 1888. Six rats underwent intracranial recordings (Song et al., 2016). The areas for craniotomy were selected based on sLORETA-based source locations at different phases of predominant IED subtypes (Bae et al., 2015). Seizure-like activities were observed during long periods of intracranial recording in several rats, but not in others. Brain slices containing any signature of a probe insertion were not included in the histological analysis.*

### IED Classification

For each electrode, IED spikes (20–70 ms duration) and sharp-waves (70–200 ms duration) were identified using a thresholding method and multiple band-stop filters (**Figure 1A**, *top-center*), and further classified into subtypes by their temporal courses and scalp topographic maps. To that end: (a) We used a three-way PARAFAC with orthogonality and non-negativity constraints, where the EEG data *V^e×t×s^* was decomposed into the superposition of atoms (each formed by a tensor product of electrode *e*, time *t*, and sample *s* signatures) and a Gaussian noise *E^e×t×s^*, i.e., $V^{e\times t\times s}=\Sigma f_{i}^{e}⊗f_{i}^{t}⊗f_{i}^{s}+E^{e\times t\times s}$, and (b) the sample signatures *S* were used to separate the IEDs into subtypes by means of the k-mean clustering method (**Figure 1A**, *atom-based 3D plots*). The representative time courses for one of the sharp-wave and spike clusters are presented (**Figure 1A**, *left*). These time courses were obtained using the mean value (X) for each atom.

### IED Localization and Brain Sectioning

To calculate the lead field needed for BSI (**Figure 1B**, *bottom left*), we co-registered the EEG minicap and the T2-weighted probabilistic atlas for Wistar rats (Valdés-Hernández et al., 2011) using the method reported in our previous study (Bae et al., 2015). Segmented and triangulated surfaces (i.e., cortex, inner/outer-skull, scalp) from the T2-weighted probabilistic atlas were used to represent those conductivity inhomogeneities introduced by different biological tissues inside the rat’s head through the use of a boundary element method (BEM) (Valdés-Hernández et al., 2016). EEG scalp topography maps (**Figure 1B**, *top-right panels*) constructed at 2/3 of the IED peak (**Figure 1A**, *vertical red-lines on the time series*) were used to estimate the cortical source distribution (i.e., sLORETA inverse solutions, Brainstorm, Pascual-Marqui, 2002; Bae et al., 2015) for each IED subtype (**Figure 1B**, *middle panels*). Since our T2-weighted probabilistic atlas is in the Paxinos and Watson’s coordinate system (i.e., referred to the Bregma location), we were able to accurately determine the block limits on an extracted brain for immunohistological evaluation where both the target region (T) and the contralateral healthy control region (C) were comprised (**Figure 1B**, *bottom-right*). We introduced a scaling factor α to correct the significant shrinking caused by the particular antigen-retrieval protocol described in the following section:

$$\alpha =\frac{L}{\left(a-p\right)}$$

The parameters *a* and *p* are the most anterior (*a* = 5.6 mm) and poste the Paxinos and Watson’s coordinate system. The parameter *L* is the actual length of the entire brain measured using the scaled brain matrix (black solid vertical lines, **Figure 1B**, *bottom-left*). Therefore, distances from the most anterior part of the cortex to Bregma (B_2_) and from the latter to the center of the brain block of interest (*l*) can be easily determined using the scaling factor. The estimated scaling factor for all rats was α = 0.741 ± 0.007 (mean ± SD). The co-registered atlas (*bottom-center*) was used to determine the particular brain regions (**Tables 1**, **2**).

**Table 2:** Convention used to identify cortical structures according to the Paxinos-Watson’s brain atlas for Wistar rats (Valdés-Hernández et al., 2011).

Region	Right	Left	Region	Right	Left
AID	1	49	PtPD	25	73
AIP	2	50	PtPR	26	74
AIV	3	51	RSD	27	75
Apir	4	52	RSGb	28	76
Au1	5	53	RSGc	29	77
AuD	6	54	S1	30	78
AuV	7	55	S1BF	31	79
Cg1	8	56	S1DZ	32	80
Cg2	9	57	S1DZ0	33	81
DI	10	58	S1FL	34	82
DIEnt	11	59	S1HL	35	83
DLEnt	12	60	S1J	36	84
DLO	13	61	S1Sh	37	85
Ect	14	62	S1Tr	38	86
Fr3	15	63	S1ULp	39	87
GI	16	64	S2	40	88
GIDI	17	65	TeA	41	89
LPtA	18	66	V1	42	90
M1	19	67	V1B	43	91
M2	20	68	V1M	44	92
MEnt	21	69	V2L	45	93
MPtA	22	70	V2ML	46	94
PRh	23	71	V2MM	47	95
PtPC	24	72	VIEnt	48	96


### Immuno-Histology

The immunohistochemical staining of brain tissue can be compromised by factors such as time of tissue dissection, type of fixation, and storage time in fixative. If fixed for longer times, the antigens are masked by crosslinking of proteins by aldehyde over-fixation. Proteolytic enzyme pre-digestion is often used to unmask the antigens, but the results are not satisfactory with over-fixed tissue. Shi et al. (1991) described a method of antigen retrieval from formalin-fixed, paraffin-embedded human brain sections based on microwave heating in metal solutions. After this initial paper, a considerable number of authors have been using the technique in different forms, i.e., **heat sources** (e.g., *microwave*, *hot plate*) and **“in solution”** (e.g., *aluminum chloride*, *citric acid*). It has been demonstrated that AR, which is simple and inexpensive, leads to adequate immunohistochemistry staining results in formalin-fixed tissue for a great number of antibodies tested (Yamashita, 2007; D’Amico et al., 2009; Shi and Taylor, 2010). As a result of all these efforts, existing formalin-fixed archival tissue collections are gaining new value. Here, we adapted this protocol to process the entire brain of rats, which has been fixed for about 6–12 months. The microwaved-version of the AR in aluminum chloride was combined with standard snap-freezing/cryostat-based sectioning with cryoprotection and Triton X-100 treatment for permeabilization. This procedure was critical to preserve brain anatomy, with the exception of the above-referred shrinking.

#### Fixation and Tissue Processing

Brains were dissected and preserved in fixative (4% paraformaldehyde) for about a year. Prior to immunohistochemical staining, brain tissues were removed from fixative and washed with distilled water for 3–4 h. Following washing, brains were incubated in 4% aluminum chloride solution at room temperature for about 12 h before heating them in a microwave (720 W) for 10 min, with a 1-min interval after the first 5 min. The 1-min interval is required to avoid brain damage from too much heat. Dimensions of each brain were measured after heating to calculate a scaling factor to compensate for shrinking of the brain upon heating (details above). Brain tissue was kept in 30% sucrose solution at 4°C for cryoprotection prior to the sectioning. Brain blocks in the freezing media were snap-frozen in liquid nitrogen and brain slices were obtained with a thickness of 30 μm. While sectioning, the coronal coordinates at which each section was taken were tracked by thickness and number of sections from anterior-to-posterior direction.

#### Immunohistochemical Staining

Sections on glass slides were rehydrated with PBS. The endogenous peroxidase activity was suppressed by incubating them in 3% hydrogen peroxide and 0.2% Triton X-100 in PBS for 30 min. Sections were washed and incubated in 3% bovine serum albumin in PBS for 1 h to block non-specific antibody binding to the tissue before incubation in primary antibodies, which was followed by secondary antibodies for 1 h each. Both the primary and secondary antibodies were diluted in PBS. We used three categories of focal epilepsy specific immunohistochemical biomarkers, namely, anatomical, inflammatory, and functional.

##### Anatomical biomarkers

For neurofilaments staining, sections were incubated for 1 h at room temperature with the primary antibody, mouse anti-neurofilament (SMI-311, 1:2000, Covance). Sections were then washed and incubated with the secondary antibody, Bovine anti-mouse IgG (sc-362246, 1:400, SantaCruz Biotech.). For Nissl staining, sections were incubated for 1 h with Neurotrace stain (N-21480, 1:300, Molecular Probes).

##### Inflammatory biomarkers

Sections were incubated for 1 h at room temperature with one of the following primary antibodies: rabbit anti-IL-1β (sc-7884, 1:300, SantaClauz Biotech.), goat anti-TNF-α (sc-1350, 1:300, SantaCruz Biotech.), and rabbit anti-HMGB1 (ab79823, 1:500, abcam). Sections were washed and incubated with respective secondary antibodies: donkey anti – rabbit IgG (Alexa Fluor 680, 1:1000, Life Technologies), donkey anti-goat IgG (sc-362255, 1:300, SantaClauz Biotech.), and donkey anti – rabbit IgG (Alexa Fluor 680, 1:1000). For glia staining, sections were incubated for 1 h with primary monoclonal mouse Anti-Glial Fibrillary Acidic (GFAP) Protein-Cya3 (C9205, 1:500, Sigma–Aldrich).

##### Functional biomarkers

Sections were incubated for 1 h at room temperature with one of the following primary antibodies: rabbit anti-NMDAR2B (AB1557P, 1:500, abcam), goat anti-mGluR-5 (sc-47147, 1:300, SantaClauz Biotech.), rabbit anti-GABAA Receptor alpha6 (AB 5610, 1:300, Millipore). Sections were washed and incubated with respective secondary antibodies: donkey anti-rabbit IgG (Alexa Fluor 680, 1:1000), donkey anti-goat IgG (sc-362255, 1:300), donkey anti-rabbit IgG (Alexa Fluor 680, 1:1000).

Coronal sections were imaged with a deconvolution microscope (Delta Vision Elite, Applied Precision) and a confocal microscope (FV-10, Olympus). Raw images were processed using the background subtraction feature in ImageJ open source software (NIH website). Number of Nissl-stained neuronal somas, perivascular cuffing densities, and number of neuronal dendritic processes were counted using the cell counter plug-in within the ImageJ software. Maximum intensity projections of background subtracted SMI-311 Z-stacks were used to model neuronal processes with high levels of contrast through the IMARIS suite (Bitplane).

Perivascular cuffing is defined as continuous vasculature-like structures densely surrounded by astrocytes (largest GFAP-stained structures). Expression of all other epilepsy specific biomarkers were computed as contiguous stained areas in an image after applying frequency threshold (ImageJ software) to avoid detecting background signal. For each rat, six images (20X) from the neocortical area of interest (T or C) were considered for the quantitative analysis of each biomarker. For this study, we did have the suitable technology for the evaluation of layer-specific cell density disorganization. First, the type of antibodies used and the image resolution at 20X were not ideal for layer delineation. Second, the random locations on the cortical sheet of these irritative zones and the region-specific inhomogeneities in individual cortical layer thicknesses impeded the use of atlases to delineate them. Therefore, these (∼600 μm × 600 μm) images were taken randomly covering all cortical layers, which provides statistical averages of cellular/molecular densities across all cortical layers in the irritative zones. For each irritative zone (i.e., the target area T), the **contralateral healthy side** was selected as a **control** (**C**). This strategy helps avoid biases caused by known inhomogeneities of cell/protein densities across the entire brain. We verified that no IED-related brain sources were visible in the control areas. In this way, target and control images were collected from each rat brain. Based on the distribution of the data, statistical analyses were performed using a paired *t*-test (for normally distributed data) and Wilcoxson’s Signed Rank test (for not normally distributed data).

#### Whole-Brain Brightfield Images

Stitched mosaic images (9) of the entire brain were generated for each irritative zone using 70 individual images (10X, FV-10, Olympus) arranged in a 10 × 7 matrix. The cortical thickness was measured in 10 randomly selected points on target and control regions of each of these stitched mosaic images. We used five different irritative zones in this analysis. We also searched for the presence of nodules in these composed images. A Wilcoxon signed-rank test was used to compare cortical thickness in target vs. control areas.

## Results

### Source Localization

For each of the seven epileptic rats, cortical sLORETA solutions were obtained for all IED subtypes. **Table 1** summarizes all irritative zones detected for each rat according to the Paxinos and Watson’s (Valdés-Hernández et al., 2011) cortical area identifiers (**Table 2**). The final target areas (structures) for each rat were selected based on two criteria: (a) their cortical source distributions were maximal (activation) for the most frequent IED subtypes and (b) its corresponding contralateral areas were silent for all IED subtypes (**Table 1**). Having a healthy contralateral cortical region is one of our inclusion criteria for the selection of the irritative zones of interest. As can be noted from **Table 1**, both M2-*right* (20) and M2-*left* (68) of rat 1884 contains irritative zones, giving the impression that the selected irritative zone (M2-*right*) does not fulfill that inclusion criterion. However, the M2 region is very long in the rostral-caudal direction. The irritative zone in M2-*right* was located on its caudal aspect (Bregma = -0.63 mm), while the one in M2-*left* was located more rostral (Bregma = +4.45 mm). Therefore, the contralateral area to the IED brain source in M2-*right* was healthy (**Supplementary Figure S1**).

Our focus is on the evaluation of anatomical, functional and inflammatory biomarkers in irritative zones that participate in the genesis of highly frequent IEDs, but that have a very low chance to be the actual seizure-onset zones. Based on previous experience, we believe these rats have only one primary seizure-onset zone. The seizure-onset zone for rats 1888 and 1894 was on the left M1 (**Table 1**), which is relatively far from their respective target areas (1888 – V2MM/S1DZ; 1894 – V2ML). Rat 1900 has the seizure-onset zone on the right AuD, which is also distant from the selected target region (S1DZ) in this rat. The selected target area for rat 2223 did not display seizures. Therefore, based on LFP recordings, the target areas in these four rats have a very low probability of being actual seizure-onset zones although they do participate in the IED genesis and propagation. LFP recordings from rat 1882 and 2225 were inconclusive as to whether the selected target areas were epileptogenic or not, since seizures were not observed during long LFP recording from the respective craniotomies. Rat 1884 did not undergo brain surgery due to a weak health condition as a result of periodic severe seizures. However, we concluded that target regions in these three rats was not the primary seizure-onset zone after examining EEG data obtained while these rats were having seizures (Bae et al., 2015). Therefore, the results from the histological analysis presented henceforth were obtained using irritative zones that were not participating in ictogenesis.

### Anatomical Markers

**FIGURE 2:**
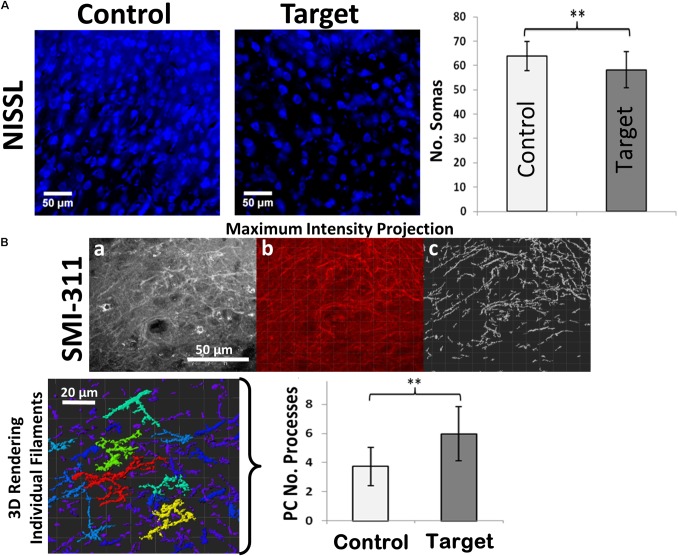
Neuronal loss and dendritic branching. **(A)** Immunofluorescence staining of Nissl-stained neuronal somas (blue) showing neuronal loss in target compared to healthy control areas. **(B)** SMI-311 staining shows neurofilament overexpression and neurons with tortuous processes in target areas. (a) A maximum intensity projection of a single SMI-311 stack demonstrates the diverse interwoven processes of local neurons. (b) A blended rendering is generated in the IMARIS suite in order to highlight the internal shadowing generated by the image depth. (c) Finally, the IMARIS suite is utilized to model processes with high levels of contrast, allowing for rapid visualization of filament density within the slice. The technique also simplifies the process of visualizing individual structures, which IMARIS suite can then color-code based on the quantity of sub-branches in the 3D rendering of individual filaments incorporating the entire Z-stack (*bottom-left*). Scale bars: 50 μm. Graphs showing the number of neuronal somas and number of neuronal processes in target and control (contralateral hemisphere) areas. Values represented as mean ± SD (*N* = 8). Significant differences referred as: ^∗^*P* < 0.05 (Paired *t*-test) and ^∗∗^*P* < 0.01 (Paired *t*-test). These representative images were taken covering layers II-IV.

We found neuronal loss (decreased number of somas) to be indicated by a reduced number of Nissl-stained neuronal somas (**Figure 2A**) in target areas compared to the corresponding (pair-wise) control areas (*p*-value < 0.01). Overexpression of SMI-311 positive neurofilaments is another important feature in FCD (**Figure 2B**). These neurofilaments allowed us to verify the presence of pyramidal cells (PCs) with dendritic processes that appeared denser (*p*-value < 0.01) and more tortuous in target areas in comparison with those in the corresponding control areas.

### Inflammatory Markers

**FIGURE 3:**
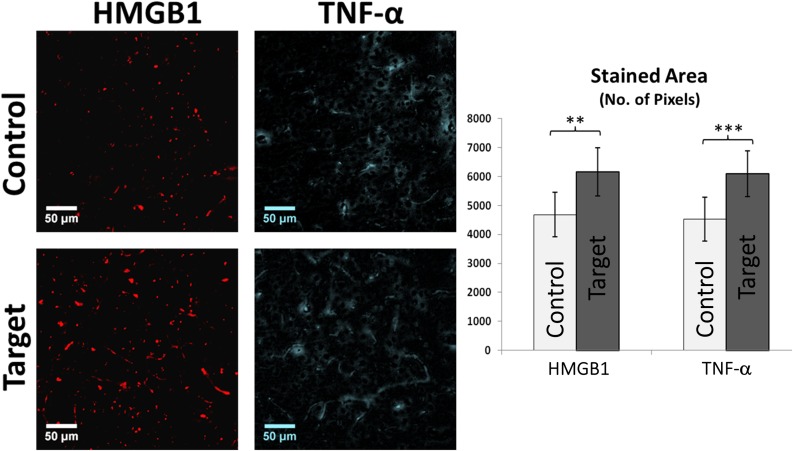
HMGB1 and TNF-α Immunofluorescence. Graph showing the overall stained area of HMGB1 and TNF-α expression indicating a clear inflammatory process in target areas compared to control. Scale bars: 50 μm. Values represented as mean ± SD (*N* = 8). Significant differences referred as: ^∗∗^*P* < 0.01 (Paired *t*-test) and ^∗∗∗^*P* < 0.001 (Paired *t*-test). These representative images were taken covering layers II-IV.

Neuroinflammation in FCD can be identified by the presence of specific inflammatory agents such as high mobility group protein 1 (HMGB1), tumor necrosis factors alpha (TNF-α), and interleukin 1-beta (IL-1β) in cortical tissue, which might trigger further immune response. HMGB1 and TNF-α are secreted by immune and swelling cells as an indicator of cell death during inflammatory processes. Increased expression of these inflammatory biomarkers in irritative zones were demonstrated by comparing target and control areas (**Figure 3**, HMGB1: *p*-value < 0.05; TNF-α: *p*-value < 0.01).

**FIGURE 4:**
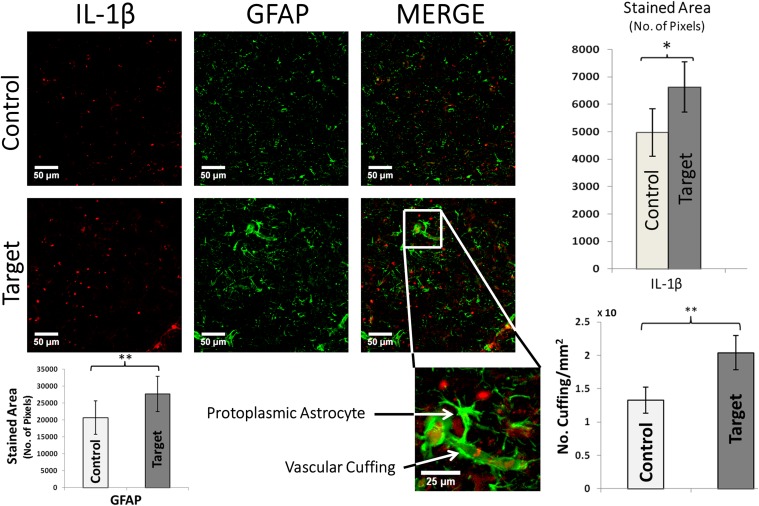
IL-1β and GFAP double immunofluorescence. Graphs (top-right) show the overall stained area of IL-1β expression in target and control areas, which indicate an undergoing inflammation in target areas. Scale bars: 50 μm. Values represented as mean ± SD (*N* = 8). Significant difference referred as: ^∗^*P* < 0.05 (Wilcoxson’s Signed Rank test). A typical enlarged protoplasmic astrocyte with end-feet attached to a micro-vessel forming frequent perivascular cuffing is shown in target area. Scale bars: 25 μm. Graphs (bottom-right) showing the density of perivascular cuffings in target and control areas. Values represented as mean ± SD (*N* = 8). Significant difference referred as: ^∗∗^*P* < 0.01 (Paired *t*-test). GFAP overexpression was quantified by computing the overall stained area of glial processes (*p*-value < 0.01, Paired *t*-test, bottom-left). These representative images were taken covering layers II-IV.

GFAP is a useful marker to demonstrate increased glial activity and the presence of astrocytes which are abnormal in their size and orientation in pathological cortical regions with FCD (**Figure 4**). The hypertrophied astrocytes and increased GFAP staining in target areas were evidence for extensive gliosis. The GFAP expression was quantified by computing total stained area of glial processes (*p*-value < 0.01, **Figure 4**, *bottom-left*). We found an abundant presence of protoplasmic astrocytes whose end-feet were in contact with and surrounded the blood vessels. Overexpression of IL-1β (**Figure 4**, *top-right*) in target areas indicate an ongoing inflammation, mainly around the blood brain barrier (BBB). We observed numerous diffuse and patchy areas of infiltration of the cortex and white matter by inflammatory cells, predominantly glia, defined as perivascular cuffing. Sections from the target area showed severe perivascular cuffing (*p*-value < 0.05, **Figure 4**, *bottom-right*) demonstrated by the presence of glial cells around an inflamed vasculature. The hypertrophied astrocytes and increased GFAP staining in target areas were evidence for extensive gliosis.

### Functional Markers

**FIGURE 5:**
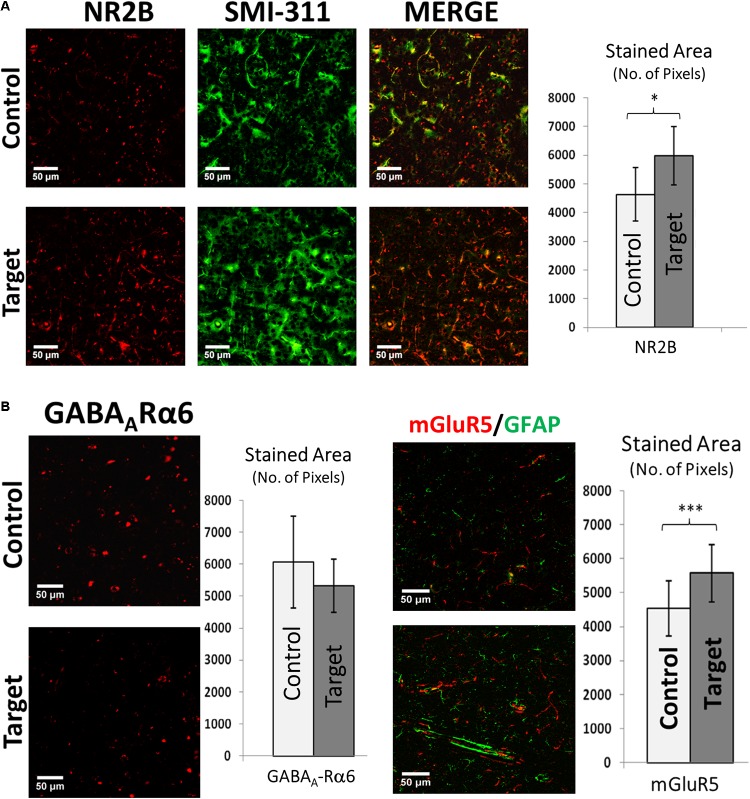
**(A)** Double immunofluorescence of NR2B and SMI-311. Double immunofluorescence showing increased expression of NMDA receptor sub-unit 2B (NR2B) and its co-expression with neurofilaments (SMI-311) in target areas indicating electrically hyper-excitable tissue. Scale bars: 50 μm. Graph showing the stained area of NR2B expression in target and control areas. Values represented as mean ± SD (*N* = 8). Significant difference referred as: ^∗^*P* < 0.05 (Wilcoxson’s Signed Rank test). **(B)** GABA_A_-Rα6 and mGluR5 expression. Left: Immunofluorescence showing the expression of Gamma-Aminobutyric Acid (a principle inhibitory neurotransmitter) receptor alpha 6 (GABA_A_-Rα6). Although there is an increase in mean GABA_A_-Rα6 expression it was not significant (*P* > 0.05) (Paired *t*-test). Scale bars: 50 μm. Values represented as mean ± SD (*N* = 8). Right: Double immunofluorescence showing increased expression of metabotropic glutamate receptor 5 (mGluR5) in target area indicating increased signaling responsible for hyper-excitability and its little co-expression with GFAP in both target and control areas indicating probable dominant neuronal source of mGluR5s compared to astroglial cells. Scale bars: 50 μm. Graph showing the stained area of mGluR5 expression in target and control areas. Values represented as mean ± SD (*N* = 8). Significant difference referred as: ^∗∗∗^*P* < 0.001 (Paired *t*-test). These representative images were taken covering layers II–IV.

Cortical activity depends on spontaneous equilibrium between excitation and inhibition. Hyper-excitability associated with irritative zones might result from larger expression of the NR2B subunit of the *N*-methyl-D-aspartate glutamate receptor (NMDR) and group I metabotropic glutamate receptor (mGluR5). Amplified expression of the NR2B receptor, related to increased glutamatergic activity, was found in target areas (*p*-value < 0.05, **Figure 5A**, *right*), which was highly co-localized with neurofilaments in neuronal soma and dendritic processes. We did not find significant differences in the target areas on the expression of the gamma-aminobutyric acid (GABA) receptor α-6 subunit (**Figure 5B**, *left*). Statistical significance may be achieved for this particular test should variability introduced by the used immunohistochemistry method (e.g., non-specific background and false-positive staining) be reduced. We observed elevated expression of mGluR5 (*p*-value < 0.001, **Figure 5B**, *right*) in target areas. mGluR5 was primarily expressed in neurons, as evident from the lack of co-localization with GFAP, although some areas showed some level of co-localizations.

### Cortical Thickness and Nodules

**FIGURE 6:**
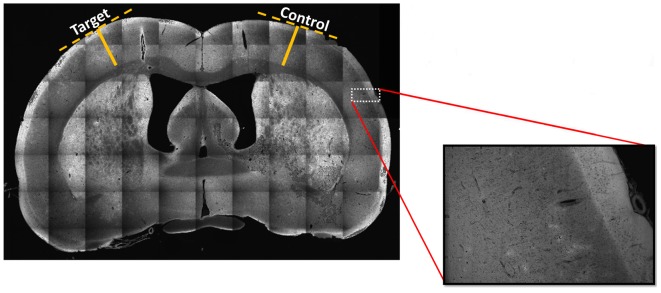
Stitched mosaic scan at 10x magnification of a representative brain slice. The stitched mosaic image is generated using 70 individual images arranged in a 10 × 7 matrix. In order to assess whether the estimated irritative zones were co-localized or not with dysplastic nodules or with areas of cortical thinning, we conducted a scan of each sagittal tissue slice at 10x magnification to quantitatively assess any cortical thinning or dysplastic nodule presence. Inset magnification of original image demonstrates such a mosaic images have the needed spatial resolution for nodule identification. Solid yellow lines indicate distances from white matter to the edges of cortex, determined using perpendicular lines (dashed, yellow) along the cortical surface. Five different irritative zones were considered for the analysis. The cortical thickness was measured in ten randomly selected points on target and control regions of nine different brain sections within each of the five irritative zones. Images were co-registered to the corresponding Paxinos and Watson’s atlas (Valdés-Hernández et al., 2011).

No significant differences were found in the cortical thickness (mean ± SD) of target (irritative zones, 1.64 ± 0.47 mm) and control (1.73 ± 0.37 mm) regions (i.e., critical/measured test statistics of *Z* = 3/Z = 1 at the significance α = 0.1, Wilcoxon signed-rank test). The Wilcoxon Two-Tailed Signed-Rank test was chosen due to its non-parametric design and its utility for conducting a paired difference test, which was ideal for the hemisphere comparison scheme. To illustrate the methodology used, a particular case is shown (**Figure 6**). The high spatial resolution of these *stitched mosaic images* (inset) allowed exploring the presence of nodules in the target and control areas. In general, nodules were not necessarily associated with irritative zones (target areas).

## Discussion

### BSI Analysis on Preclinical Models of Focal Epilepsy

In this study, we applied the BSI methodology (Bae et al., 2015; Valdés-Hernández et al., 2016) to identify brain regions spontaneously generating IEDs in rats with FCDs and used them for postmortem histological evaluation. We were successful in performing immunostaining for three categories of specific biomarkers for focal epilepsy with fluorescence immunohistochemistry in a brain fixed for a few months to a year. Rodents are widely used these days to study epilepsy. They have also been very useful to create preclinical models of other brain disorders, e.g., Alzheimer’s disease (Ashe and Zahs, 2010), stroke (Mergenthaler et al., 2004), autism (Umeda et al., 2010) and schizophrenia (Shevelkin et al., 2014). BSI in rodents will help localize brain sources associated with specific electrophysiological abnormalities in these disorders. However, it is challenging to record EEG data from these small animals with the spatial resolution required to perform BSI. In this study, we proved this concept by applying the BSI method to localize the irritative zones of chronic epileptic rats in the same way it is done in humans.

#### Anatomical Biomarkers

We found that irritative zones in FCD were characterized by the existence of abnormal neurons overexpressing neurofilaments. Consistent results were reported previously in dysplastic compared to non-dysplastic areas in human cases of type IIA and IIB FCD (Finardi et al., 2013). These irritative zones showed larger number of neuronal processes with increased tortuosity, as well as reduced neuronal density demonstrated by Nissl staining similar to those found in their dysplastic cortical regions (Thom et al., 2005). Neuronal loss, which was not found either in temporal lobe epilepsy (TLE) with hippocampal sclerosis nor in mild malformation or cortical development, might be associated with either neuropil expansion, local failures in neuronal migration/proliferation, or secondary effects. Increases in dendritic lengths and tortuosity have been reported in the past in the hippocampus of patients with TLE (da Silva et al., 2006). As far as we know, these features have not been associated with FCD. Neurons showing thick and large somas have been associated with sustained epileptiform-like activity in FCD (Colciaghi et al., 2011), which might be responsible in forming abnormal cortical networks giving rise to hyper excitability (Cepeda et al., 2003).

#### Inflammatory Biomarkers

Recent evidence strongly suggests that glia-mediated inflammation plays a role in the pathogenesis of seizures and epilepsy (Devinsky et al., 2013). We found extensive reactive gliosis (with altered astrocyte morphology) in localized irritative zones which corroborates finding that longer chronic epilepsy is associated with more intensive reactive gliosis and astrocyte alterations in epileptogenic region, further evidencing that FCD epilepsy is a progressive pathology (Finardi et al., 2013). Astrocytic processes have been found to be swollen under osmotic and ischemic stress (Risher et al., 2009). The activation of astrocytes includes changes that vary with the severity and chronicity of seizure pathology (Sofroniew, 2009). Reactive astrocytes has been reported in animal models of epilepsy and in neural tissue from human patients with mesial temporal sclerosis (MTS), FCD, tuberous sclerosis complex (TSC), Rasmussen’s encephalitis, and glioneuronal tumors (Devinsky et al., 2013). Astrocytes are closely related to the microvasculature and with their end-feet ensheathing blood vessels known as perivascular cuffing contribute to BBB function (Devinsky et al., 2013). We observed increased perivascular cuffings and inflammatory glial nodules in irritative zones of the rat epileptic brain. These structures have been attributed in the past to tissue breakdown associated with frequent seizures (Rasmussen et al., 1958).

Pro-inflammatory chemokines and cytokines released by inflamed epileptogenic tissue can interact with their respective receptors, overexpressed by brain microvasculature in epilepsy, thus compromising BBB permeability at multiple levels. Glial cells play a critical role by maintaining balance between pro-inflammatory and anti-inflammatory pathways. Immune system and glia including astrocytes release IL-1β and HMGB1 which activate nuclear factor kappa B (NF-κB), an important regulator of pro-inflammatory gene expression which has been reported to be upregulated in MTS and TSC tissue (Crespel et al., 2002; Maldonado et al., 2003). In the case of IL-1β and HMGB1 release, signaling occurs through activation of the pro-inflammatory IL-1 receptor/toll-like receptor (IL1R/TLR) system (Maroso et al., 2010). Our results also showed increased TNF-α staining in the target area with lack of co-localization with GFAP, indicating presence of pro-inflammatory immune response with non-astrocytic sources of TNFα.

In *in vivo* seizure models, astroglial cells are also key sources of anti-inflammatory molecules such as the IL-1 receptor antagonist (IL-1ra), an endogenous competitive IL-1 receptor blocker that controls IL-1β-mediated inflammation. IL-1ra has powerful anticonvulsant effects in experimental seizure models (Vezzani et al., 2000) and mice overexpressing IL-1ra in astrocytes were reported to be intrinsically resistant to seizures (Vezzani et al., 2000). However, in MTS and experimental epilepsies, astrocyte expression of IL-1ra is significantly lower than that of IL-1β (Ravizza et al., 2006), which may explain poor anti-inflammatory response due to the dominance of pro-inflammatory pathways over anti-inflammatory pathways which we demonstrate to be consistent with our finding of IL-1β over expression in epileptogenic regions. Also, pro-inflammatory IL-1β was found to be co-localized with GFAP at perivascular cuffing locations, indicating active inflammation. This supports previous studies which reported astrocyte derived IL-1β compromising BBB integrity during seizures (Librizzi et al., 2012). Seizures can induce alterations in the expression of IL-1β and TNFα (Vezzani and Granata, 2005). Here, we demonstrated that these alterations are not only limited to the seizure-onset zones.

#### Functional Biomarkers

NMDA receptors (NMDAR) are likely to play an important role in epileptogenesis because the NMDAR channels are permeable to Ca^2+^ which acts as a secondary messenger in signaling attributed to synaptic plasticity (Kirkwood et al., 1993). Crino et al. (2001) reported increased NR2B mRNA in dysplastic neocortical neurons in human cortical dysplasia. In relevance to these findings, we demonstrate elevated NR2B expression in localized irritative zones with more intense co-localization of NR2B subunits with neurofilaments in neuronal soma and neuronal dendritic processes.

So far, it is clear that cortical injuries are associated with abnormal excitability of the adjacent neuronal networks in focal epilepsy. It has been assumed that the development of this abnormal neuronal activity is the result of an imbalance between excitation and inhibition, where changes in inhibition are believed to play the most important role (Imbrosci and Mittmann, 2011). Functionally, the impaired GABAergic transmission has been attributed to a reduction in the release of GABA from presynaptic terminals and changes in the expression of GABA receptors (Calcagnotto et al., 2005; Imbrosci and Mittmann, 2011). We found a reduction, although not statistically significant, in GABAA-receptor-α6 subunit of GABA receptor family in these irritative zones. To reach a proper conclusion in this regard, we need to analyze the expression profiles of the entire GABA receptor family and improve our immunohistochemistry technique while also taking into account previous studies (Schiene et al., 1996) reporting differential GABAergic transmission in and around epileptic foci in epileptogenic cortex.

Activation and high expression of group I metabotropic glutamate receptors (mGluRs) may contribute to neuronal hyperexcitation, as suggested by the convulsing action of group I agonists, which are responsible for seizure discharge and epileptogenesis in different experimental models (Wong et al., 1998). In our study, we observed elevated expression of mGluR5 (*p*-value < 0.001) in irritative zones with the dominant source being neuronal as evident from lack of co-localization of mGluR5 with GFAP. Since consistent findings were reported in similar studies involving resected FCD tissue, glioneuronal tumors and dysembryoplastic neuroepithelial tumors from clinical patients associated with chronic intractable epilepsy which are highly epileptogenic (Aronica et al., 2001, 2003), we can speculate that chronic seizure activity could contribute to the strong expression of mGluR5 in neuronal components in the cortical dysplasia although the role of group I mGluRs is not yet completely understood (Bruno et al., 1999).

A characterization of the layer-dependent cellular/molecular disorganization in the cortical dysplastic regions (lesions/nodules) of this particular focal epilepsy model has been previously done (Colciaghi et al., 2011; Pennacchio et al., 2015). For the first time, our data shows that similar cellular/molecular density abnormalities are also found in the irritative zones of these rats. Although the cortical dysplastic regions we found in these rats resemble typical characteristics of those reported for this FCD preclinical model, it was difficult to demonstrate any consistent connection with the network of irritative zones.

## Conclusion

In brain irritative zones, we found abnormal anatomical structures (i.e., larger neuronal processes, glioreactivity, and vascular cuffing) and higher expression of neurotransmission (NR2B) and inflammation (i.e., ILβ1, TNF α and HMGB1) biomarkers. While it still has not been entirely proven that these irritative zones are overlapped with seizure-onset zones, a significant quantity of literature attests to the oftentime overlap between these regions. Due to the high level of concordance in the abnormal profiles for the irritative and seizure-onset zones, the former holds the possibility of becoming future epileptogenic areas, but this pattern is not always consistent and further longitudinal studies are required to understand the transition dynamics. Through the BSI methodology, we believe that it may become possible to localize abnormal brain tissues in FCD patients that are prospective for being epileptogenic before becoming seizure-onset zones, which will have implications in the development of novel preventive and therapeutic methods.

## Ethics Statement

We confirm that we have read the Journal’s position on issues involved in ethical publication and affirm that this report is consistent with those guidelines. All experimental procedures in this study were approved by our Institutional Animal Care and Use Committee (IACUC). The protocol number is 13-004.

## Author Contributions

AD and JL collected the immunohistological data and the image processing. YS performed the intracranial recording. YS and JB obtained the EEG scalp recordings. PV-H assisted us with the EEG forward/inverse problem analysis. W-CL participated in all discussions and helped with the data analytics. AD and JR wrote the paper. JR designed and conducted the entire study.

## Conflict of Interest Statement

Cortech Solution’s has licensed a patent (US 9,078,584 B2) from Tohoku University (Sendai, Japan) for the EEG mini-cap. JR might receive 10% of the patent-generated royalties. The other authors declare that the research was conducted in the absence of any commercial or financial relationships that could be construed as a potential conflict of interest. The reviewer MSA and the handling Editor declared their shared affiliation.

## Supplementary Material

FIGURE S1 Illustration of the M2 region for Wistar rats (green-left and red-right). Note that it distributes along the rostral-caudal direction. The irritative zone in M2-*right* was located on its caudal aspect (Bregma = -0.63 mm), while the one in M2-*left* was located more rostral (Bregma = +4.45 mm).
