# Supplementary figures and images for: Histological Characterization of the Irritative Zones in Focal Cortical Dysplasia Using a Preclinical Rat Model
Source: Front Cell Neurosci. 2018 May 18;12:52. doi: 10.3389/fncel.2018.00052 (PMC5968101; doi:10.3389/fncel.2018.00052)

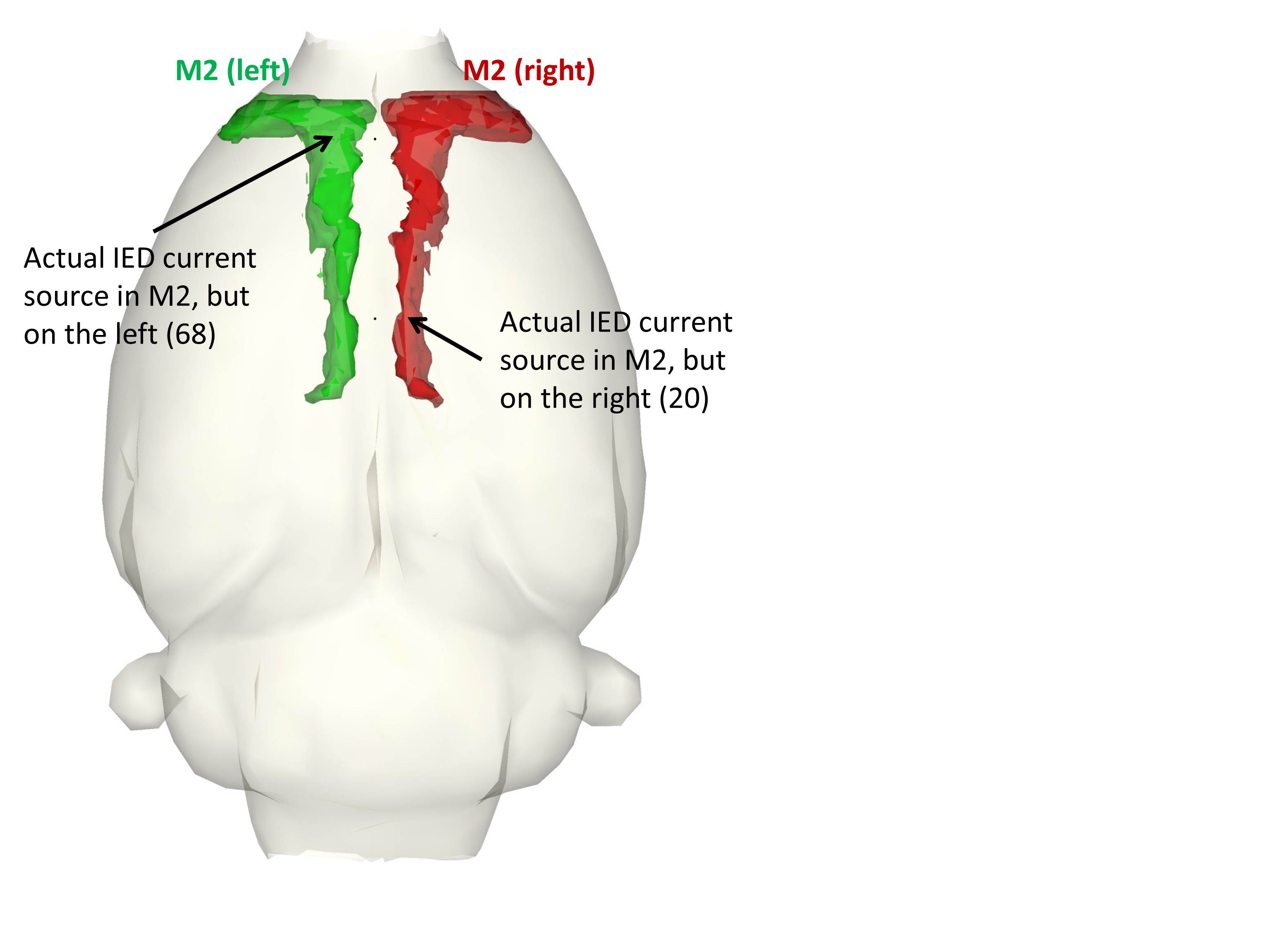

Supplement: FIGURE S1 — Illustration of the M2 region for Wistar rats (green-left and red-right). Note that it distributes along the rostral-caudal direction. The irritative zone in M2-right was located on its caudal aspect (Bregma = -0.63 mm), while the one in M2-left was located more rostral (Bregma = +4.45 mm). [file Image_1.JPEG]
